# Investigation of the actual implementation of “post‐sedation discharge criteria” and “time‐out” immediately before procedure in endoscopy: A nationwide survey study in Japan

**DOI:** 10.1002/deo2.70149

**Published:** 2025-05-27

**Authors:** Atsushi Imagawa, Motohiko Kato, Junko Koyama, Mitsuhiro Fujishiro

**Affiliations:** ^1^ Department of Gastroenterology Imagawa Medical Clinic Kagawa Japan; ^2^ Center for Diagnostic and Therapeutic Endoscopy, Keio University School of Medicine Tokyo Japan; ^3^ Endoscopy Center, Tochigi Cancer Center Tochigi Japan; ^4^ Department of Gastroenterology Graduate School of Medicine the University of Tokyo Tokyo Japan

**Keywords:** endoscopy | patient discharge | perioperative care | sedation | time‐out

## Abstract

**Objectives:**

Post‐sedation discharge criteria for outpatient endoscopy and time‐out procedures immediately before endoscopic examinations are important for ensuring patient safety. This study used a web‐based questionnaire to survey the implementation status and current situation of these practices in Japan in 2024.

**Methods:**

A self‐administered questionnaire was conducted from December 2023 to January 2024 using Google Forms. Participants were primarily from facilities involved in endoscopy study groups and readers of an endoscopy‐specific e‐newsletter. Additionally, medical staff from endoscopic centers across Japan were invited to participate in collaboration with the Japan Gastroenterological Endoscopy Technicians Society.

**Results:**

A total of 1,495 valid responses (medical staff: 1197 [80%]; doctors: 298) were collected from 1168 facilities, after excluding duplicate responses. Among the participating facilities, 58% were general hospitals, 21% were clinics or health check‐up centers, and 9% were university hospitals or national cancer centers. Post‐sedation discharge criteria were implemented in 58% of facilities for esophagogastroduodenoscopy and 56% for colonoscopy, with the post‐sedation recovery score used as the criterion in about half of these cases. Time‐out procedures were implemented in 57% of the facilities for both esophagogastroduodenoscopy and colonoscopy. Items confirmed during time‐out in more than half of the facilities included: patient's name, details of antithrombotic drugs, content of examination, drug allergies, underlying disease, date of birth, consent form, age, procedure start time, and patient's identification number.

**Conclusion:**

The implementation rate of post‐sedation discharge criteria and time‐out procedures was found to be close to 60%, reflecting the real‐world situation in Japan in 2024.

## 1 Introduction

In Japan, gastrointestinal endoscopy—including esophagogastroduodenoscopy (EGD) and colonoscopy—is widely performed as a routine examination at various facilities, ranging from private clinics to university hospitals. These procedures are conducted on a broad spectrum of patients, from adults undergoing screening to elderly individuals with high‐risk conditions and comorbidities. Meanwhile, sedation is increasingly used to reduce anxiety and resistance to the procedure while enhancing patient satisfaction. Despite the diverse settings in which these procedures are performed, standard criteria typically exclude an assessment of psychomotor performance. Moreover, as the scoring systems are designed for postoperative surgery rather than endoscopy, and given the variety of available systems, standardized discharge criteria following sedated endoscopy have not yet been established [1]. Additionally, the use of propofol, which is commonly administered abroad, is restricted under Japanese medical insurance, limiting sedation options to benzodiazepines. Since the types of benzodiazepines used are not standardized, sedatives with varying half‐lives are administered, often leading to confusion in medical settings. To avoid such confusion, uniform discharge criteria are essential.

In recent years, significant progress has been made in endoscopic procedures beyond routine examinations, with the development of complex techniques such as endoscopic submucosal dissection [2] for early‐stage gastrointestinal cancers and invasive biliary drainage [3]. As a result, perioperative management during and after sedation has become increasingly important. An online survey by the European Society of Gastrointestinal Endoscopy found that 56.3% of facilities have implemented discharge criteria [4]. Additionally, a study of 50 teaching facilities in Korea reported that all had introduced discharge criteria [5]. However, despite the development of various discharge criteria, universally applicable and easily implementable guidelines have yet to be established [6].

For medical staff in endoscopy centers, numerous checks must be performed before and after procedures to assess patient status and ensure safety during treatment. Checklists in surgery have been systematically established and include the following: (1) sign‐in, to verify the patient's background upon arrival in the operating room; (2) time‐out, to confirm procedural details with all staff immediately before the operation; and (3) sign‐out, to review the patient's condition, operative results, and resection specimen before leaving the operating room. The implementation of this system has been effective in reducing mortality and complications [7, 8]. In gastrointestinal endoscopy, sign‐in and sign‐out have primarily been adopted for therapeutic endoscopic procedures, similar to their use in surgery. However, the need to introduce time‐outs for enhanced safety has only recently been recognized [9, 10, 11, 12, 13, 14, 15]. Although systematic reviews have shown that checklists contribute to improved team communication and procedural standardization, their direct impact on patient safety, such as reducing complications, has been limited [9, 10, 14]. Conversely, discharge criteria are believed to play a key role in ensuring patient safety by supporting smooth and safe discharge after sedation [16]. In routine outpatient endoscopy, patients come from diverse backgrounds, making it necessary to implement discharge criteria to prevent unexpected complications after sedation and to introduce time‐out procedures to reduce procedural risks. In Japan, a 2019 report found that discharge criteria had been adopted in 65% of advanced centers, while time‐out had been implemented in 61% of centers [17]. Since 2016, the Study Group for Standardization of Periprocedural Management for Gastrointestinal Endoscopy has been meeting semiannually to promote the implementation of discharge criteria and time‐out procedures. However, no survey had been conducted to assess the current adoption rates nationwide, including private clinics and general hospitals. To assess the current situation in Japan in 2024, 5 years after the previous survey, a questionnaire was distributed to a large number of facilities with 2500 doctors and 16,000 medical staff.

## 2 Methods

### 2.1 Design and study population

A self‐administered questionnaire was conducted from December 2023 to January 2024 using Google Forms. The primary respondents were physicians affiliated with the Japan Gastroenterological Endoscopy Society (JGES), as well as members of the following study groups on endoscopy: the Study Group for Standardization of Periprocedural Management for Gastrointestinal Endoscopy, the Fight‐Japan Group, and the Madowazu Group. Additionally, readers (endoscopists) of an endoscopy‐specific e‐newsletter participated in the survey. Furthermore, in collaboration with the Japan Gastroenterological Endoscopy Technicians Society (JGETS), medical staff from endoscopy centers across Japan were also invited to respond.

### 2.2 Survey development

The survey was developed by the authors, with items generated based on a review of the literature and the research team's clinical experience.

### 2.3 Data analysis

For each participant, the following background data were collected: the size of the participating facility, the annual number of outpatient EGD and colonoscopy procedures, the types of sedatives and analgesics primarily used, the implementation rate of discharge criteria, and the type of criteria adopted. Regarding time‐out, participants were asked about its implementation rate and the specific checklist items used. Open‐ended responses were also collected. Questions about the types of sedatives and analgesics were only posed to facilities that used them. Similarly, additional questions regarding the type of discharge criteria and the specific items used in time‐out were only asked in facilities where these measures had been introduced. The web‐based questionnaire consisted of a maximum of 20 items, and the estimated time required to complete it was approximately 3–4 min. The survey, distributed via a personal mailing list to collect diverse opinions, resulted in multiple responses from some facilities. To ensure data validity, representative responses were selected based on predefined criteria. In cases where multiple doctors or medical staff from the same facility responded, the following criteria were applied to select a representative response:

When responses were identical, one response was selected.

When responses differed, one response per facility was selected based on the following priority order:

- The most frequently reported response.
- Responses from medical staff were prioritized over those from endoscopists, as endoscopists’ responses were primarily personal.
- Responses with names were given priority (providing a name was optional).

### 2.4 Statistical analysis

Descriptive statistics were performed using Microsoft Excel (Microsoft Corporation, Redmond, Washington). Categorical data were presented as numerical values, with percentages depicted in brackets.

## 3 Results

**FIGURE 1:**
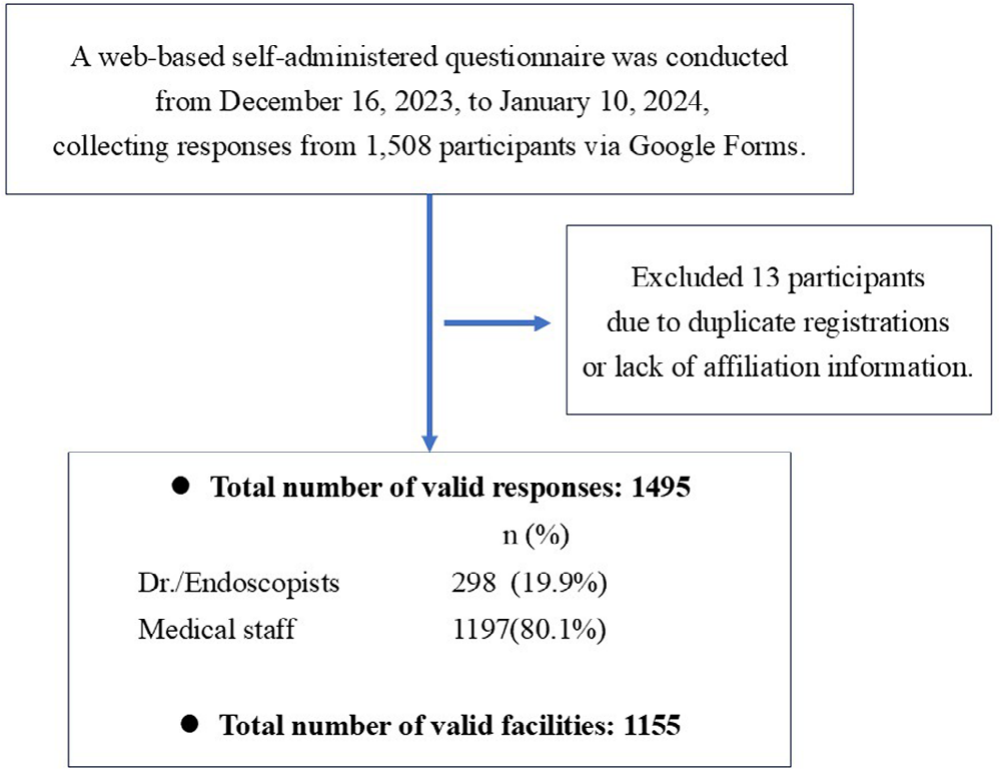
Flow diagram of survey respondents via Google Forms. A total of 298 (19.9%) respondents were endoscopists and 1197 (80.1%) were medical staff. The total number of valid facilities after removing duplicate responses was 1155.

A total of 1508 responses were received via Google Forms. Of these, 13 were excluded due to duplicate registrations or unknown affiliations, resulting in 1495 valid responses. Among them, 298 (19.9%) were endoscopists, and 1197 (80.1%) were medical staff. After removing duplicate responses using the method described in the data analysis, the number of unique responding facilities was 1155 (Figure 1). Among these facilities, 249 (21.6%) were private clinics, 102 (8.8%) were university hospitals or national cancer centers, and 804 (69.6%) were general hospitals (Table 1). Additionally, 738 facilities (63.9%) performed more than 1000 EGD procedures per year, while 496 facilities (43.4%) conducted more than 1000 colonoscopies annually. Responses were thus obtained from centers with a high procedural volume (Table 2).

**TABLE 1:** Distribution of participating facilities.

Number of facilities	*n* (%)
Private clinic	249 (21.6)
Hospitals with less than 500 beds	676 (58.5)
Hospitals with more than 500 beds	128 (11.1)
University hospitals and the National Cancer Center	102 (8.8)

**TABLE 2:** Distribution of annual number of endoscopies at participating facilities.

Number of annual endoscopies	EGD 1155 *n* (%)	Colonoscopy 1143^†^ *n* (%)
5000−	210 (18.2)	52 (4.5)
1000–4999	528 (45.7)	444 (38.8)
100–999	383 (33.2)	517 (45.2)
1–99	34 (2.9)	130 (11.4)

Abbreviation: EGD, esophagogastroduodenoscopy.

^†^ Colonoscopy not performed at 12 facilities.

### 3.1 Sedatives and analgesics

Since midazolam is approved for endoscopic sedation under medical insurance in Japan, sedatives were used in 90% of facilities, with midazolam serving as the primary sedative in nearly 70% of facilities for both EGD and colonoscopy. In contrast, propofol, which is commonly used in Europe and the United States, was administered in approximately 3% of facilities. Sedatives were used in half of the facilities for EGD and in three‐quarters of facilities for colonoscopy. Regarding analgesics, pentazocine was the most frequently used for EGD, while pethidine was the most commonly used for colonoscopy (Table 3).

**TABLE 3:** Survey results of sedation and post‐sedation discharge criteria for esophagogastroduodenoscopy and colonoscopy.

	EGD	Colonoscopy
Number of facilities	1155	1143^†^
	*n* (%)	*n* (%)
**Mainly used sedative**		
Unused	79 (6.8)	117 (10.2)
Midazolam	791 (68.5)	792 (69.3)
Flunitrazepam	45 (3.9)	44 (3.8)
Diazepam	75 (6.5)	49 (4.3)
Propofol	37 (3.2)	32 (2.8)
Other^‡^	128 (11.1)	109 (9.5)
**Mainly used analgesics**		
Unused	610 (52.8)	296 (25.9)
Pethidine	214 (18.5)	424 (37.1)
Pentazocine	274 (23.7)	356 (31.1)
Other^‡^	57 (4.9)	67 (5.9)
**Introduction of “post‐sedation discharge criteria”**		
Already installed	674 (58.4)	643 (56.3)
Not installed	367 (31.8)	371 (32.5)
Other/no answer	114 (9.9)	129 (11.3)
Number of facilities already installed		
Post‐sedation discharge criteria	674	643
**Evaluation Methods for post‐sedation discharge criteria**		
PSRS^§^	336 (49.9)	318 (49.5)
Score original to the facility	231 (34.3)	220 (34.2)
Aldrete score	74 (11.0)	72 (11.2)
MPADSS	8 (1.2)	8 (1.2)
PADSS	3 (0.4)	3 (0.5)
Other/no answer	22 (3.3)	22 (3.4)

Abbreviations: EGD, esophagogastroduodenoscopy; PSRS, post‐sedation recovery score; MPADSS, modified post‐anesthesia discharge scoring system; PADSS, post‐anesthesia discharge scoring system.

^†^ Colonoscopy not performed at 12 facilities.

^‡^ Including multiple‐dose use.

^§^ Developed by the Japan Gastroenterological Endoscopy Technicians Society.

### 3.2 Discharge criteria

The rate of discharge criteria implementation was similar between EGD and colonoscopy, with 674 facilities (58.4%) adopting them for EGD and 643 facilities (56.3%) for colonoscopy. The post‐sedation recovery score an original assessment tool developed by JGETS, was the most widely used, implemented in 49.9% of facilities for EGD and 49.5% for colonoscopy. This score evaluates a combination of consciousness, motor function, and vital signs, making it relatively easy to implement (Tables 3 and 4) [18].

**TABLE 4:** Post‐sedation recovery score.

Categories	Points
**Level of consciousness**	
Clearly responds to verbal stimulation	2
Awakens in response to verbal stimulation but cannot maintain awakening	1
No response to verbal stimulation	0
**Motor function**	
Able to move limbs freely and walk without unsteadiness	2
Able to move limbs but with limited range of motion	1
Unable to move limbs freely	0
**Respiratory status**	
Able to take deep breaths and cough freely	2
Dyspnea or tachypnea is observed	1
Apneic state is observed	0
**Circulatory dynamics**	
Systolic BP >100 mmHg or recovered to pre‐sedation levels	2
Systolic BP <50% decrease compared to pre‐sedation levels	1
Systolic BP >50% decrease compared to pre‐sedation levels	0
**Oxygen saturation**	
SpO_2_ >92% without oxygen supplementation	2
Requires oxygen supplementation to maintain SpO_2_ >90%	1
Even with oxygen supplementation, SpO_2_ recovers to < 92%	0

*Note*: A total patient's score of 10 points is considered fit for discharge home.

Abbreviations: BP, blood pressure; SpO_2_, peripheral oxygen saturation.

### 3.3 Time‐out

Time‐out procedures were implemented in 653 of 1155 facilities (56.5%) for EGD and in 647 of 1143 facilities (56.6%) for colonoscopy. The items confirmed during time‐out for both procedures included the patient's name, details for antithrombotic drugs, content of examination, drug allergies, underlying disease, date of birth, consent form, patient's age, procedure start time, and patient's identification number (Table 5).

**TABLE 5:** Survey results of time‐out for esophagogastroduodenoscopy and colonoscopy.

	EGD	Colonoscopy
Number of facilities	1155	1143^†^
	*n* (%)	*n* (%)
**Introduction of “time‐out”**		
Already installed	653 (56.5)	647 (56.6)
Not installed	412 (35.7)	415 (36.3)
Other/No answer	90 (7.8)	81 (7.1)
**Check items in “Time‐out” items at installed facilities**		
Number of facilities	653	647
	*n* (%)	*n* (%)
Patient's name	645 (98.8)	633 (97.8)
Details for antithrombotic drugs	524 (80.2)	530 (81.9)
Content of examination	477 (73.0)	475 (73.4)
Drug allergies	473 (72.4)	466 (72.0)
Underlying disease	395 (60.5)	415 (64.1)
Date of birth	393 (60.2)	389 (60.1)
Consent form	378 (57.9)	386 (59.7)
Patient's age	328 (50.2)	335 (51.8)
Procedure start time	140 (21.4)	155 (24.0)
Patient's identification number	30 (4.6)	30 (4.6)

Abbreviation: EGD, esophagogastroduodenoscopy.

^†^ Colonoscopy not performed at 12 facilities.

## 4 Discussion

In Japan, only one report, based on a 2019 survey and released in 2022 (with an English abstract), has examined the introduction rates of discharge criteria and time‐out. A study of 66 leading facilities found that both discharge criteria and time‐out after sedation had been implemented in approximately 60% of facilities [17]. In the present study, Google Forms was used to collect real‐world data from facilities across Japan, covering more than 1000 institutions, ranging from private clinics to high‐volume centers and university hospitals. The results revealed that discharge criteria were implemented in 58% of facilities for EGD and 56% for colonoscopy, while time‐out procedures were introduced in 57% of facilities for both procedures, providing an updated overview of the current situation in Japan as of 2024. Although the adoption rates for discharge criteria and time‐out procedures were slightly lower than in a previous survey conducted 5 years ago, the inclusion of a broader range of facilities, from private clinics to general hospitals, suggests that these measures have become widely accepted throughout Japan.

Regarding sedatives, midazolam was used in more than 70% of facilities due to restrictions on insurance coverage in Japan. Due to the narrow range of effective and appropriate blood concentrations and the risk of respiratory depression, propofol administration in Japan is restricted to anesthesiologists and other qualified professionals. While the use of ultrashort‐acting propofol requires caution, its application has been increasing in therapeutic endoscopy with the support of Bispectral Index monitoring and target‐controlled infusion systems [19, 20]. However, propofol has been introduced in only about 3% of routine screening procedures. The current situation highlights the difficulty of implementing non‐anesthesiologist administration of propofol in Japan.

Although discharge criteria have been reported as problematic due to insufficient evaluation of their usefulness [21], they are recognized as helpful tools for facilitating patient discharge and ensuring outpatient safety. In clinical practice, both patients and medical staff feel greater peace of mind when discharge criteria are implemented [16, 22, 23]. In Korea, all endoscopy units adhered to the Accreditation of Qualified Endoscopy Unit program of the Korean Society of Gastrointestinal Endoscopy (KSGE), which was introduced in 2012 to promote systematic quality management [5]. In contrast, Japan and Europe have lower adoption rates (around 60%), likely due to the wider survey range and limited evidence supporting these measures [4]. Nonetheless, implementing a standardized check system and improving staff training to ensure patient observation remain important priorities in Japan. Currently, discharge criteria and time‐out procedures are voluntary measures in Japan, which may contribute to lower implementation rates due to the perceived increase in workload. Establishing national guidelines or standardized protocols could help promote adoption, ultimately improving patient safety and reducing the burden on medical staff. As the use of sedatives continues to increase, and with the anticipated future introduction of remimazolam—currently unavailable in Japan—it would be ideal to establish a nationally standardized discharge scoring system [16, 24–26]. While the modified Post‐anesthesia discharge scoring system and the Aldrete score are commonly used as discharge criteria overseas [27, 28, 29, 30, 31, 32, 33] their adoption rate in Japan remains low, with some facilities developing and implementing their own scoring systems [34]. The post‐sedation recovery score (Table 4), developed specifically for Japan, evaluates five categories (consciousness, motor function, respiratory status, circulatory dynamics, and oxygen saturation), each scored on a three‐point scale (0‐1‐2), for a total of 10 points. If the score is below 10, the patient should continue to be monitored until reaching 10 points, which is considered a safe discharge threshold. Given that discharge criteria are often first assessed by medical staff, this simple scoring system is likely to become more widely adopted. A key issue with discharge criteria is the absence of clear evaluation methods, highlighting the need for further validation regarding their practicality and effectiveness. Future research should assess the frequency of post‐examination complications and the satisfaction levels of both patients and medical staff. Additionally, guidelines vary across countries regarding the recommended observation period, and a standardized duration has not yet been established. In some facilities, securing sufficient personnel and space in the recovery room presents challenges, making the ideal two‐hour observation period difficult to implement in practice.

The introduction of time‐out in endoscopy in Japan is still relatively new, and some confusion persists in clinical settings. This is due to overlapping sign‐in checklist items, variations in time‐out procedures across facilities, unclear distinctions between time‐out and sign‐in, and the absence of uniform standards. Additionally, in high‐volume centers, the occasional occurrence of patients with identical names and surnames undergoing endoscopy on the same day underscores the need for verifying detailed information, such as the patient's date of birth and identification number, to prevent misidentification. To enhance efficiency, it is crucial to focus on verifying only essential details within a short time, similar to preoperative time‐out in surgery. Key elements should include confirming the presence or absence of antithrombotic drugs and reviewing the examination details. Time‐out procedures also help improve communication among staff and are believed to contribute to higher procedural quality and safety. However, further research is needed to establish their effectiveness in gastrointestinal endoscopy.

There are some limitations in this study. Although this survey was conducted in more than 1000 facilities, the results may be somewhat overestimated, as responses were primarily provided by medical staff from facilities that actively participated in the survey. In this survey, questionnaires were distributed to approximately 2500 endoscopists and 16,000 medical staff nationwide. Responses were received from 1495 individuals (response rate: 8.1%). Although the response rate was low, personal email addresses were used, and some may have been inactive. Notably, 363 of the responding facilities (24.3%) were designated training or affiliated training facilities of the JGES (total:1499 facilities)[35]. Thus, we consider the results to be reasonably reliable. Another limitation is the discrepancy between the responses of endoscopists and medical staff within the same facilities, which may have affected data accuracy. While objective data were unavailable, it appeared that the responses from medical staff were more representative of the actual situation than those from endoscopists. This is because the endoscopists generally offered personal opinions, while medical staff were more likely to reflect the overall facility perspective in their responses. Many comments were received from medical staff in the free‐response section. Some of the most concerning remarks indicated a lack of support from endoscopists for the implementation of discharge criteria and time‐out procedures. Although medical staff at many facilities are strongly committed to enhancing safety protocols, the reluctance of some endoscopists to adopt these measures remains a significant challenge, as they are often occupied with daily responsibilities and may not fully recognize the importance of post‐sedation patient management. The nationwide adoption of discharge criteria and time‐out procedures is essential for enhancing patient safety and comfort during endoscopic examinations. Ultimately, ensuring safe and comfortable procedures will lead to increased examination uptake and facilitate the early detection of malignant tumors.

In conclusion, this study has provided insight into the real‐world implementation of discharge criteria and time‐out procedures in Japan. It is hoped that adoption rates will continue to rise and that nationally standardized criteria will be developed in the future.

## Conflicts of Interest

The authors declare no conflicts of interest.

## Ethics Statement

The present study was approved by the ethics committee of the Kagawa Medical Association (No.2023‐08) on December 15, 2023.

## Consent

Written consent was not required because this study was a web‐based survey of endoscopy systems at various facilities, rather than a study involving patients or diseases.

## Clinical Trial Registration

Not applicable

## Data Availability

All data and study materials from this study will be made available to other researchers.
